# Supplementary figures and images for: Cross-cohort generalizability of deep and conventional machine learning for MRI-based diagnosis and prediction of Alzheimer’s disease
Source: Neuroimage Clin. 2021 Jun 4;31:102712. doi: 10.1016/j.nicl.2021.102712 (PMC8203808; doi:10.1016/j.nicl.2021.102712)

Supplementary files

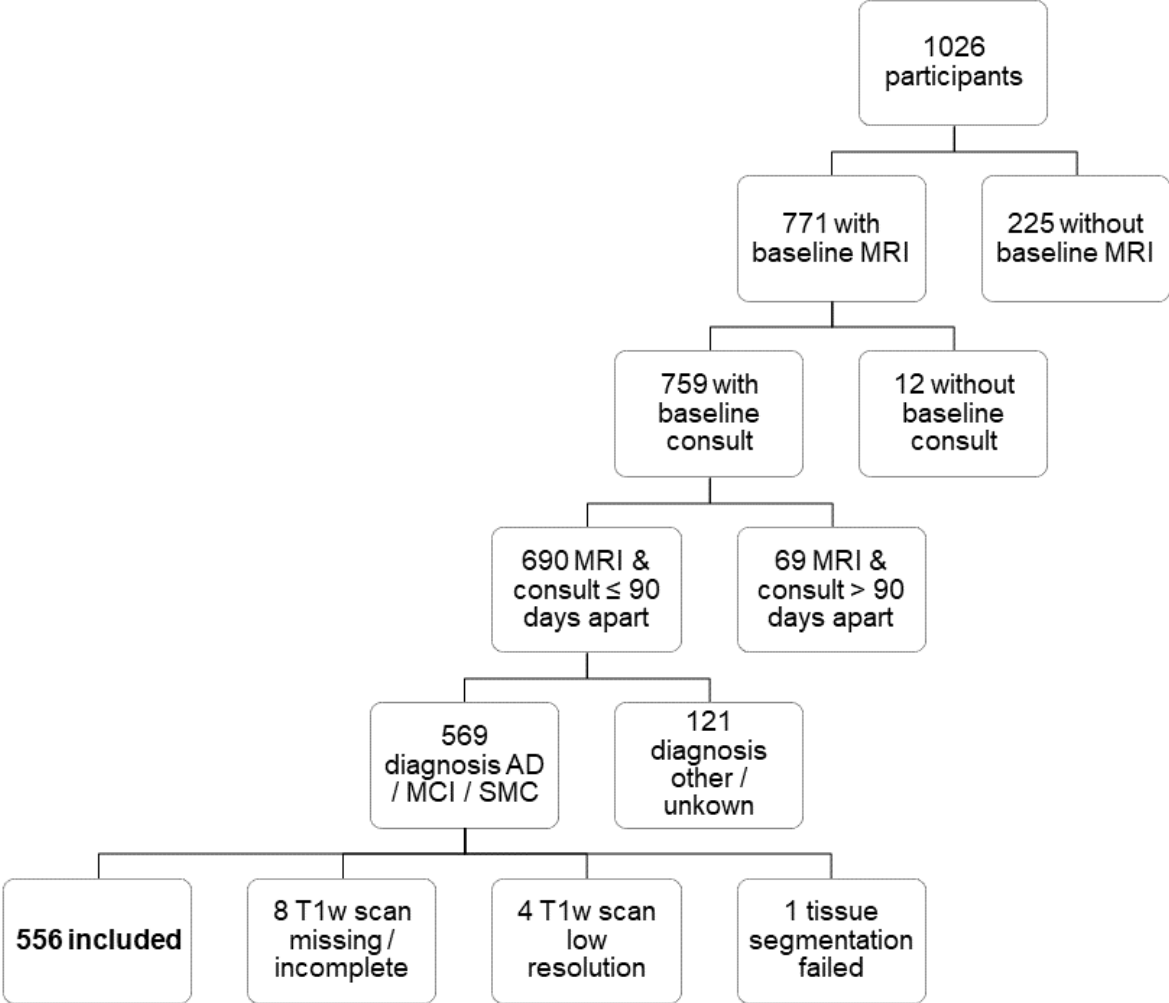

Figure S1: Inclusion of participants of the PND data set

Supplement: Supplementary data 1 [file mmc1.pdf]
